# Model of a 3D Magnetic Permeability Tensor Considering Rotation and Saturation States in Materials with Axial Anisotropy

**DOI:** 10.3390/ma16093477

**Published:** 2023-04-29

**Authors:** Dominika Kopala, Anna Ostaszewska-Liżewska, Peter Råback, Roman Szewczyk

**Affiliations:** 1 Institute of Metrology and Biomedical Engineering, Faculty of Mechatronics, Warsaw University of Technology, sw. A. Boboli 8, 02-525 Warsaw, Poland; anna.lizewska@pw.edu.pl (A.O.-L.);; 2 CSC–IT Center for Science, P.O. Box 405, FI-02101 Espoo, Finland; peter.raback@csc.fi

**Keywords:** soft magnetic materials, uniaxial magnetic anisotropy, tensor of magnetic permeability

## Abstract

The paper proposes a 3D extension of the linear tensor model of magnetic permeability for axially anisotropic materials. In the proposed model, all phases of a magnetization process are considered: linear magnetization, magnetization rotation, and magnetic saturation. The model of the magnetization rotation process is based on the analyses of both anisotropic energy and magnetostatic energy, which directly connect the proposed description with physical phenomena occurring during a magnetization process. The proposed model was validated on the base of previously presented experimental characteristics. The presented extension of the tensor description of magnetic permeability enables the modelling of inductive devices with cores made of anisotropic magnetic materials and the modelling of magnetic cores subjected to mechanical stresses. It is especially suitable for finite element modelling of the devices working in a magnetic saturation state, such as fluxgate sensors.

## 1. Introduction

Modelling the behaviour of inductive components with magnetic cores in numerous mechatronic devices is based on the description of relative magnetic permeability ***μ***, which is one of the most important properties of magnetic materials. For simple isotropic systems, relative magnetic permeability ***μ*** can be approximated by a scalar value. However, such a description is insufficient in the case of the most common technical applications [1], such as electrical steels [2] or amorphous alloys [3]. Since commonly used modern soft magnetic materials [4] are characterised by strongly anisotropic properties [5,6,7], relative magnetic permeability ***μ*** has to be described as a 3 × 3 tensor. Another problematic aspect of magnetic modelling is that such materials often work near the saturation state. This case occurs in transductors [8], pressductors [9], and other sensors using ferromagnetic cores [10]. It should be highlighted that when a magnetization process ends in a full saturation state, the directions of the magnetizing field ***H*** and the flux density ***B*** induced in the material are parallel. However, in the case of anisotropic materials, for smaller values of a magnetizing field ***H***, the angle between the magnetizing field ***H*** and the flux density ***B*** should be considered in modelling [11].

A reasonably simplified, although still faithful from the physical point of view, description of magnetic properties is necessary for modelling the inductive components with anisotropic magnetic cores. Recently, different types of software for finite element modelling offer the implementation of the tensor description of relative magnetic permeability ***μ***, such as commercial COMSOL [12] and ANSYS [13] or open-source ELMER FEM [14]. Apart from the tensor description, one must consider the magnetization process’s nonlinear characteristics related to saturation flux density *B_s_*.

Due to the need for a mathematical description of the magnetization curve, many different models have been proposed. The known models can be divided into two main categories [15], where “type I” models are constructed from the superposition of scalar models distributed along all possible directions, and “type II” are constructed by the integration of contributions of intrinsically vector elements. Since classical “type I” models are based on a scalar approach, like in the scalar Preisach model [16], they would find no use in the tensor description. Moreover, the mentioned model is limited to isotropic materials, just like many models already presented in the literature [17,18,19,20]. The use of the proposed models is impossible for modern soft magnetic materials due to their anisotropic characteristics. The “type II” models are based on different physical phenomena, such as the dry friction-like hysteresis mechanism based on domain wall pinning (B-DF model) [21], Stoner–Wohlfarth (SW) model for monodomain magnetic systems [22], or energy-based models (EB) that are derived from thermodynamic principles [23,24]. The new models are mainly the extensions of already existing ones which improve their accuracy and increase application possibilities, such as the anisotropic extension [25] of the Jiles–Atherton model [26] or the MultiScale Model (MSM) [27] based on the B-DF model. The given models deal with anisotropy modelling.

The given paper proposes another approach to magnetic curve modelling based on tensor and energy operations addressing the uniaxial anisotropy of materials. The primary solution for a given problem was proposed by introducing the tensor model of relative magnetic permeability for two-dimensional models [28]. The two-dimensional simplification is sufficient for describing the magnetic behaviour of bulk inductive components with stress-induced anisotropy. However, the lack of three-dimensional models limits the use of such a solution for bulk objects. The paper presents the new idea of a three-dimensional, linear–rotation–saturation (LRS) model of relative magnetic permeability tensor ***μ***, which considers uniaxial anisotropy for soft magnetic materials and allows for a saturation state. The model is based on the physical principles describing a magnetizing process [29], in which the saturation state is connected with the rotation of magnetic flux density vector ***B*** towards the direction of magnetizing field ***H***. The proposed model can be used for efficient and accurate modelling of three-dimensional inductive components with axial anisotropy. Moreover, since only axial anisotropy occurs in isotropic materials subjected to mechanical stresses [30], the proposed model of a magnetization process can be used for complex magneto-mechanical modelling cases where isotropic magnetic materials are subjected to axial and shear mechanical stresses described by a stress tensor ***σ***.

## 2. Axial Anisotropy of Relative Magnetic Permeability Tensor *μ* in 3D

**Figure 1:**
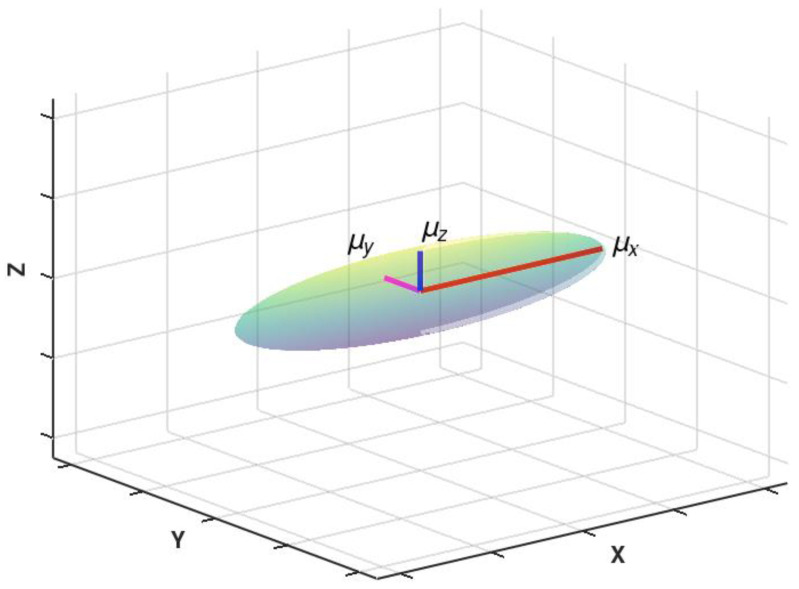
The visualisation of relative magnetic permeability tensor ***μ*** for axial anisotropy in three dimensions.

The visualisation of the three-dimensional principal axis representation of the relative magnetic permeability tensor for anisotropic magnetic materials is presented in Figure 1. The shape of the representation is an ellipsoid. Due to the uniaxial character of anisotropy, two semi-axes are the same length. These semi-axes *µ_y_* and *µ_z_* determine the permeability with respect to the hard axes *Y* and *Z* of magnetization, whereas the permeability with respect to the easy axis *X* equals *µ_x_*.

The three-dimensional principal axis description of relative magnetic permeability tensor ***μ*** is now given by

(1) $$\mu =\left[\begin{array}{ccc} \mu_{x} & 0 & 0\\ 0 & \mu_{y} & 0\\ 0 & 0 & \mu_{z} \end{array}\right]$$

It should be highlighted that for any system with axial anisotropy (with the coordinate axes not being aligned with the principal axes of the permeability tensor), the proposed description requires a 3D rotation. In such a case, a rotation matrix ***R*** can be used [31] as

(2) $$\mu_{R}=R^{-1}\mu R$$

where ***R*** can be given in various forms. In the most general case, the rotation matrix ***R*** can be given in the Euler form as an array of cosine values between the principal *XYZ* and the rotated *X’Y’Z’* coordinate axes [32]:

(3) $$R=\left[\begin{array}{ccc} \cos(X,X^{\prime }) & cos(X,Y^{\prime }) & \cos(X,Z^{\prime })\\ \cos\left(Y,X^{\prime }\right) & \cos(Y,Y^{\prime }) & \cos(Y,Z\prime )\\ \cos(Z,X^{\prime }) & \cos(Z,Y^{\prime }) & \cos(Z,Z^{\prime }) \end{array}\right]$$

As a result, all analyses presented in the paper can be easily converted to any orientation of material anisotropy by performing the rotation transformations in terms of the matrix ***R***.

## 3. The Proposed Linear–Rotation–Saturation (LRS) Model of Relative Magnetic Permeability for Materials with Axial Anisotropy

In line with the physical description [11], the proposed model considers three phases of a magnetization process:

- The linear phase.
- The magnetization rotation phase, when the length of flux density vector ***B*** is equal to saturation flux density *B_s_*; however, the magnetostatic energy is consumed for the rotation of flux density ***B*** vector towards the magnetizing field ***H***.
- The magnetic saturation phase, in which when the length of flux density vector ***B*** is equal to saturation flux density *B_s_*, it is parallel to the magnetizing field ***H***, and saturation is modelled by shrinking the magnetic permeability tensor.

To simplify the model explanation, the case of axial anisotropy is considered when the easy axis of magnetization is in line with the *X* axis. In such a case, also for high-permeability materials, in the initial linear phase of the magnetization process, the magnetic flux density ***B*** is given as

(4) $$B_{L}=\mu_{0}\left[\begin{array}{ccc} \mu_{x} & 0 & 0\\ 0 & \mu_{y} & 0\\ 0 & 0 & \mu_{z} \end{array}\right]H$$

where *μ*_0_ is the magnetic constant. In the proposed model, the length of the magnetic flux density vector ***B*** grows with the magnetizing field ***H*** growth until reaching the value of saturation flux density *B_s_*. However, when the length of flux density ***B*** reaches the magnetic saturation value *B_s_*, the material is not yet fully saturated. The second phase of the saturation process is related to the rotation of magnetic flux density ***B*** towards the direction of the magnetizing field ***H***.

**Figure 2:**
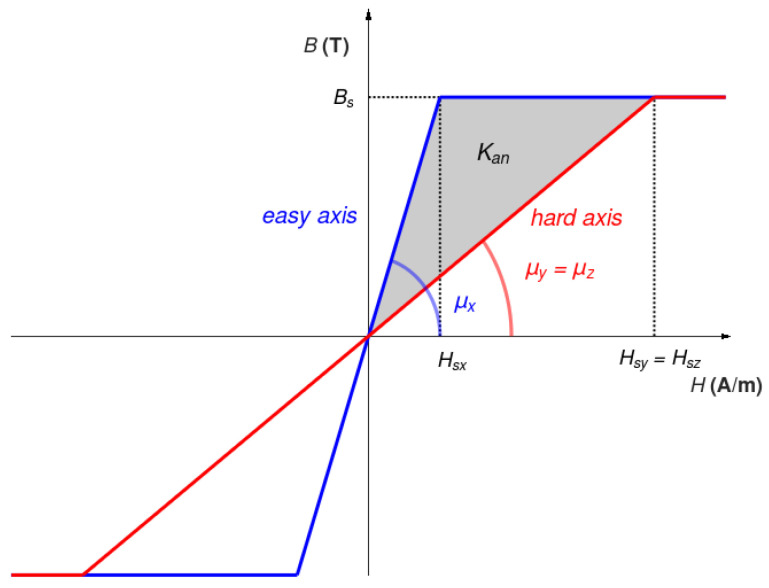
Simplified *B(H)* magnetization curves of axially anisotropic magnetic material, magnetized in the direction of the easy axis (red) and hard axis (blue) of magnetization.

The magnetization rotation phase model requires analysing the magnetostatic energy of materials by considering hard and easy magnetization curves. Moreover, in the case of stress-induced axial anisotropy, relative magnetic permeability values *µ_y_* and *µ_z_* with respect to the hard axes *Y* and *Z* of magnetization are the same [33]. In such a case, simplified *B(H)* magnetization curves (linear with saturation) are presented in Figure 2.

In order to reach full saturation, the magnetizing vector needs to move from the easy axis to the hard axis of magnetization. For this process, a specific amount of energy is required. In the simplified linear model with saturation, the energy necessary to obtain full saturation is equal to magnetostatic energy. Based on the plots presented in Figure 2, the value of magnetostatic energies *E_x_* and *E_y_* (required for the magnetic saturation in *X* and *Y* directions, respectively) can be calculated based on geometric dependencies:

(5) $$E_{x}=\frac{B_{s}H_{sx}}{2}=\frac{B_{s}^{2}}{2\mu_{0}\mu_{x}}$$

(6) $$E_{y}=\frac{B_{s}H_{sy}}{2}=\frac{B_{s}^{2}}{2\mu_{0}\mu_{y}}$$

where *B_s_* is the saturation flux density (determined by the chemical composition of the material and being equal for *X* and *Y* directions), whereas *H_sx_* and *H_sy_* are the values of the magnetizing field required for the magnetic saturation in *X* and *Y* directions, respectively. Finally, the average magnetic anisotropy energy *K_an_* (measured per cubic meter of the material) required for the rotation of magnetization in saturation from the easy axis to the hard axis of the material [34] is given as

(7) $$K_{an}=E_{y}-E_{x}=\frac{B_{s}^{2}}{2\mu_{0}}\left(\frac{1}{\mu_{y}}-\frac{1}{\mu_{x}}\right)\left[\frac{J}{m^{3}}\right]$$

In the rotation state, the flux density $B_{s}^{\prime }$ in the material can be described as

(8) $$B_{s}^{\prime }=\mu_{0}\left(R^{-1}\left[\begin{array}{ccc} \mu_{x} & 0 & 0\\ 0 & \mu_{y} & 0\\ 0 & 0 & \mu_{z} \end{array}\right]R\right)H_{s}$$

where ***R*** is the rotation matrix, and ***H_s_*** is given as

(9) $$H_{s}=H\frac{\left|B_{L}\right|}{B_{s}}$$

In order to perform the rotation of magnetic flux density ***B***, the value of required energy and the rotation angle need to be calculated. The energy can be described as the difference between the total magnetic energy connected with the magnetizing field ***H*** and the magnetic field energy required to reach saturation *B_s_*:

(10) $$E_{rot}=\frac{B_{s}\left(\left|H\right|-\left|H_{s}\right|\right)}{2}$$

The angle *ϕ* associated with the rotation is used to define the total energy *E_an_(ϕ)* required for performing this process. For a uniaxial material, their relationship can be described by a formula [11]:

(11) $$E_{an}\left(\phi \right)=K_{an}{sin}^{2}\phi$$

where *K_an_* is the average anisotropy energy density defined in Equation (7) (alternatively the anisotropy coefficient), and *ϕ* is the angle of magnetization concerning the axis of magnetization. This formula can be simplified to the linear form:

(12) Eanϕ=ϕπ2Kan

In order to define a rotation angle *φ_rot_* of ***B***, one condition needs to be checked. If the energy *E_rot_* passed for rotation is lower than the anisotropic energy *E_an_(ϕ)*, so if *E_rot_* < *E_an_(ϕ)*, the rotation angle *φ_rot_* equals

(13) $$\varphi_{rot}=\phi \frac{E_{rot}}{E_{an}\left(\phi \right)}$$

The full saturation state is reached in the case where *E_rot_* ≥ *E_an_(ϕ)*. In full saturation, the rotation angle *φ_rot_* equals the angle *ϕ* between the easy magnetization axis and the direction of the magnetizing field ***H***.

**Figure 3:**
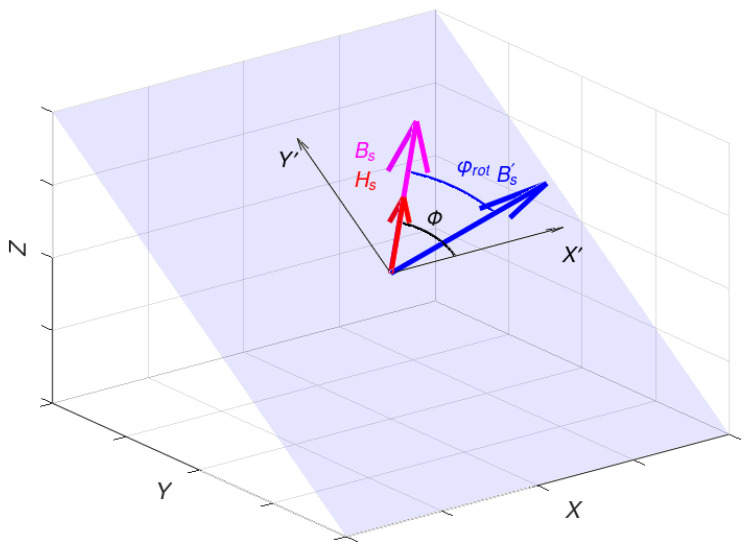
The angles between magnetizing field ***H***, easy-magnetization axis *X’*, and flux density ***B*** in the rotation plane *X’Y’* (magnetizing field H – red arrow, flux density B before rotation – blue arrow, flux density B after rotation – magenta arrow).

In the final saturation stage, the magnetic flux density ***B*** is parallel to the direction of the magnetizing field ***H***. The length of the magnetic flux density vector ***B*** equals the saturation value *B_s_* and cannot increase despite the increase in the magnetizing field ***H***. The visualisation of rotation angles in the saturation process is presented in Figure 3.

For a numerical implementation, the rotation of the saturated vector ***B*** can be calculated with the use of a three-dimensional rotation matrix ***R***:

(14) $$B=RB_{s}^{\prime }$$

where $B_{s}^{\prime }$ is given by Equation (8).

From a mathematical point of view, the three-dimensional rotation matrix ***R*** can be obtained in many ways, depending on the complexity of a rotation process. In our case, the rotation by the rotation angle *φ_rot_* causes the movement of the magnetic flux density vector ***B*** towards the magnetic field strength vector ***H***. As both vectors emanate from the origin of the *XYZ* system, both share a common plane, which can be defined by a surface normal originating from the same origin. To simplify the case, the common plane for both vectors can be defined by a unit normal ***u_n_*** (obtained by normalising surface normal), which equals the vector product of the normalised magnetic flux density vector ***u_B_*** and the normalised magnetizing field ***u_H_***, so that

(15) $$u_{n}=u_{B}\times u_{H}$$

where

(16) $$u_{B}=\frac{B}{\left|B\right|},u_{H}=\frac{H}{\left|H\right|}$$

The common plane for both vectors, where the rotation takes place, does not usually overlap with the primary coordinate system planes such as *XY*, *XZ*, or *YZ*. In practice, it is convenient to introduce a new coordinate system *X’Y’Z’* for rotation such that ***B*** and ***H*** vectors lie in the *X’Y’* plane and the unit normal ***u_n_*** is the rotation axis *Z’*. In such a way, the rotation can be performed for vectors located anywhere in the body. Thus, the rotation corresponding to the rotation angle *φ_rot_* about the rotation axis ***u_n_*** = [*u_nx_*, *u_ny_*, *u_nz_*] can be performed by using the rotation matrix defined as follows:

$$R=\left[\begin{array}{ccc} c+u_{nx}^{2}\left(1-c\right) & u_{nx}u_{ny}\left(1-c\right)-u_{nz}s & u_{nx}u_{nz}\left(1-c\right)+u_{ny}s\\ u_{ny}u_{nx}\left(1-c\right)+u_{nz}s & c+u_{ny}^{2}\left(1-c\right) & u_{ny}u_{nz}\left(1-c\right)-u_{nx}s\\ u_{nz}u_{ny}\left(1-c\right)-u_{ny}s & u_{nz}u_{ny}\left(1-c\right)+u_{nx}s & c+u_{nz}^{2}\left(1-c\right) \end{array}\right]$$

where

(17) $$s=sin\left(\varphi_{rot}\right),c=\cos\left(\varphi_{rot}\right)$$

**Figure 4:**
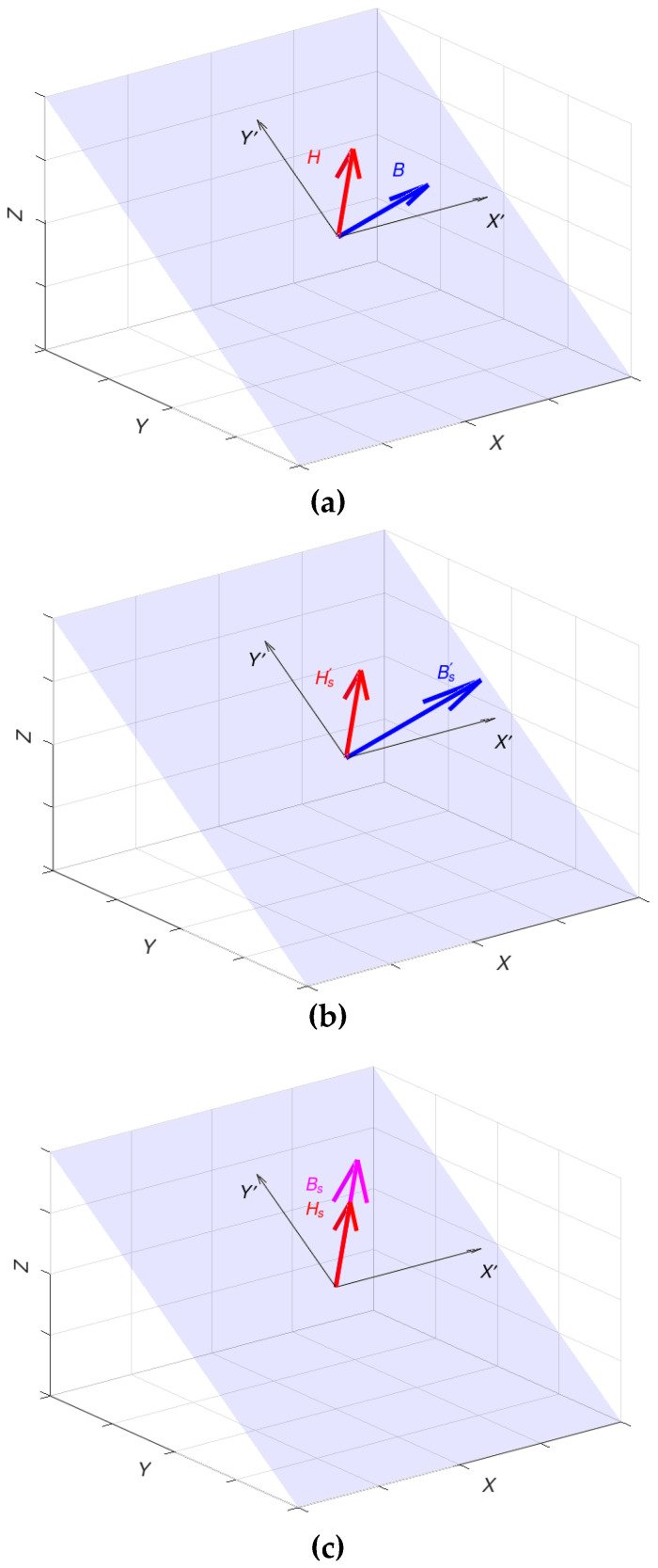
Three phases of the magnetization process for material with uniaxial anisotropy: (**a**) linear magnetization region, (**b**) material saturated—flux density vector ***B*** not parallel to magnetizing field ***H***, (**c**) fully saturated material. (magnetizing field H – red arrow, flux density B before rotation – blue arrow, flux density B after rotation – magenta arrow).

The derived rotation matrix ***R*** can be used to perform the rotation in Formula (14). The graphical summary of the proposed process needed in the linear–rotation–saturation (LRS) model is presented in Figure 4. The whole magnetization process conducted for a specific range of values for magnetic field strength ***H*** and permeability tensor components *μ_x_, μ_y_* and *μ_z_* is also presented in the Supplementary Materials as a video file.

The detailed process description is used to derive the final form of the permeability tensor in each phase. In the first linear phase (Figure 4a), where the saturation induction is not reached, the magnetic permeability tensor is described in a simple form:

(18) $$\mu_{L}=\left[\begin{array}{ccc} \mu_{x} & 0 & 0\\ 0 & \mu_{y} & 0\\ 0 & 0 & \mu_{z} \end{array}\right]$$

As noticed, the permeability values depend solely on the initial material parameters in the linear phase.

After the value of saturation induction has been reached, while, at the same time, the vectors ***H*** and ***B*** are not parallel to each other, rotation is performed. In this stage (Figure 4b), the length of ***H*** is reduced to the value *H_s_*, as presented in Formula (9). The magnetic permeability tensor must be modified using the rotation matrix in Formula (17). In this phase, the permeability tensor is described as

(19) $$\mu_{rot}=R^{-1}\left[\begin{array}{ccc} \mu_{x} & 0 & 0\\ 0 & \mu_{y} & 0\\ 0 & 0 & \mu_{z} \end{array}\right]R$$

In the final saturation state, when both the vectors ***H*** and ***B*** are aligned with the hard magnetization direction, the magnetic permeability tensor is given as follows:

(20) $$\mu_{s}=\left(R^{-1}\left[\begin{array}{ccc} \mu_{x} & 0 & 0\\ 0 & \mu_{y} & 0\\ 0 & 0 & \mu_{z} \end{array}\right]R\right)\frac{\left|H_{s}\right|}{\left|H\right|}$$

The above description can be utilised to build a numerical FEM version of the magnetic permeability model. As magnetic permeability is a property that characterises the magnetic behaviour of the material well, its accurate numerical description is a key factor affecting the accuracy of FEM models [35].

The proposed model was implemented with open-source Octave software [36]. The source files are available in the Supplementary Materials for further validation and development.

## 4. Modelling of Magnetization Curves

**Figure 5:**
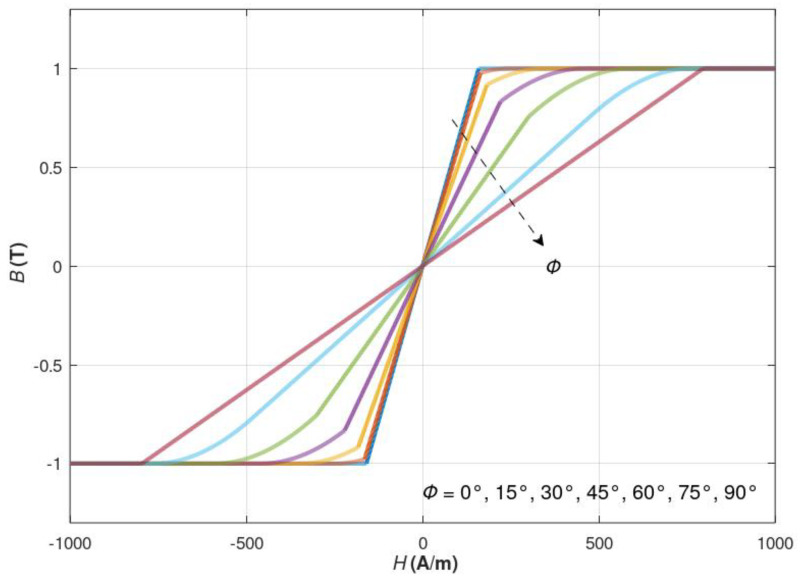
The magnetizing curves for different angles *ϕ* between magnetizing direction and easy-magnetization axis.

For the proposed method, magnetization curves *B(H)* have been modelled for different angles of the magnetizing field ***H***. The presented magnetic flux density vector ***B*** is a projection on the direction of the magnetizing field ***H***. Such a case occurs in a model where the magnetizing coil is coaxial with the sensing coil placed around the magnetic core. An example of such a model is a fluxgate sensor in Foerster configuration [37]. The magnetization curves for different angles between the magnetizing field ***H*** and the easy axis of magnetization are presented in Figure 5.

For the states where the magnetizing field ***H*** is parallel to either the minor or the major axis of the relative magnetic permeability tensor, rotation does not appear. The area between the curves for easy and hard magnetization axes represents the rotation state area, where the rotation state is represented by the plot curvature between the not-saturated and the saturated state.

The proposed model generates simplified results similar to the observation made when analysing experimental results for different amorphous magnetic alloys with uniaxial anisotropy. Such anisotropy can be

- Induced during the thermomagnetic annealing (Experiment in Ref. [38]).
- Induced as a stress-induced anisotropy in isotropic magnetic materials (such as soft ferrites) subjected to mechanical stresses (Experiments in Refs. [39,40]).

It should be highlighted that the experimentally observed results of the measurements of the characteristics of soft magnetic materials with uniaxial anisotropy are well represented by the proposed model.

**Figure 6:**
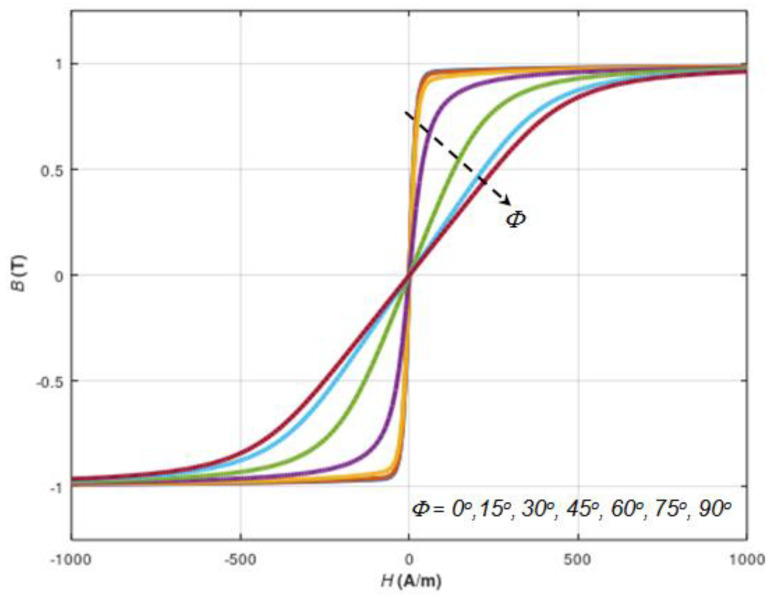
The magnetizing curves for different angles *ϕ* between magnetizing direction and easy-magnetization axis.

In addition to the measurement results, characteristics obtained from the proposed, generalised 3D linear–rotation–saturation (LRS) model can be compared to single dimension axial anisotropy magnetization curve models, enabling consideration of the angle *Φ* between the direction of the anisotropy easy axis and the direction of the magnetizing field *H*. An example of the results of modelling the magnetization curve of soft magnetic material with uniaxial anisotropy and with an extended Jiles–Atherton model [17] is presented in Figure 6.

It should be indicated that the character of changes in the magnetization curve of soft magnetic material with uniaxial anisotropy modelled with an extended Jiles–Atherton model can be well represented by the proposed generalised 3D linear–rotation–saturation (LRS) model. Moreover, the proposed LRS model generates more general results, enabling fast and robust 3D relative magnetic permeability tensor modelling. The previously presented extended Jiles–Atherton model enables only the modelling of the magnetization curve in a given direction *Φ*.

On the other hand, it should be highlighted that the proposed model is suitable only for modelling the uniaxial anisotropy of soft magnetic materials. Other models should be proposed for modelling other types of anisotropy, such as the magnetocrystalline anisotropy of hard magnetic materials or grain-oriented electrical steels.

## 5. Practical Implementation

The proposed linear–rotation–saturation (LRS) model can be implemented directly when the magnetic field strength dependence of relative permeability is used in FEM models. However, if the magnetic model utilised edge elements, magnetic potential analyses are carried out by FEM software (e.g. ELMER FEM 9.0, CSC – IT Center for Science, Espoo, Finland), and the relationship derived from the Maxwell equation [41] is utilised:

(21) $$\nabla \times \left(\nu B\right)-\sigma E^{\prime }=g$$

where *σ* is electric conductivity, *E* is the electric field, and *g* is a divergence-free source. In such a case, it is necessary to transform permeability *μ* to reluctivity *ν* as a function of flux density *B* instead of magnetic field strength *H*. The relation between those is given by

(22) $$\upsilon (B)=\mu^{-1}(B)$$

It should be highlighted that to solve Equation (22), the linear or nonlinear ***B****(**H**)* dependence should be considered, depending on the working point on the magnetization curve. On the other hand, the ***B****(**H**)* relation is always monotonous due to physical reasons. As a result, the successive approximation method can be applied to the assessment of ***ν****(**B**)* dependence.

The transition phase between the linear and rotational phase causes the ***B***(***H***) curve to have a discontinuous derivative at the transition point. Since FEM solutions are based on the methods of solving differential equations, solving the problem of discontinuous derivatives is a mathematical problem. It is solved in different ways, with both classical and numerical approaches. In our numerical case, several solutions have been provided regarding this issue [42,43]. The discontinuity problem in FEM modelling is addressed primarily in the cases of mechanical cracks [44]. The analysis should be provided individually for each modelling case in terms of convergence for discontinuities. One example is the Discontinuous Galerkin formulation [45]. FEM modelling tools enable using different techniques for solving differential equations, which should be adjusted to a specific modelling case.

## 6. Conclusions

The proposed 3D extension of the linear tensor model for axially anisotropic magnetic permeability considers three phases of a magnetization process: linear magnetization, magnetization rotation, and magnetic saturation. Moreover, the magnetization rotation process model is based on the analyses of both anisotropic energy and magnetostatic energy, which directly connect the proposed description with physical phenomena occurring during the magnetization process. The proposed rotation matrix ***R***-based description can be easily implemented into finite element software, such as open-source ELMER FEM or other commercially available alternatives. In the case of such implementation, the relative permeability tensor can be modelled, which creates new possibilities for modelling the 3D magnetostatic and magnetodynamic systems.

The modelling results align with previously presented and experimentally measured characteristics of soft magnetic materials with axial anisotropy, such as amorphous alloys subjected to annealing in the presence of a magnetic field or isotropic magnetic materials (such as soft ferrites) subjected to mechanical stresses. Moreover, the results align with other numerical models of anisotropic hysteresis. As a result, the proposed extension of the tensor description of the magnetic permeability model enables the modelling of inductive devices with cores made of anisotropic magnetic materials, together with the modelling of magnetic cores subjected to mechanical stresses, which is important for sensors working with magnetostriction effects, such as energy harvesting devices [46,47]. For this reason, the proposed model is especially suitable for finite element models of devices working in a magnetic saturation state, such as fluxgate sensors. Since the proposed model is three-dimensional, it can be applied to extend existing 2D models, such as a 2D frame-shaped fluxgate sensor [48]. In addition, due to the fact that both axial and shear stresses can be reduced to axial stresses in the principal directions [30], the proposed model might be used for modelling the axial and shear stress dependence of characteristics of fluxgate sensors with cores made of isotropic materials. This means that the influence of compressive and tensile stresses, bending, and torque should be considered in the modelling. Moreover, the stress dependences of the 3D relative magnetic permeability tensor model and the model of magnetic saturation are required. The proposed model covers all these requirements and opens up the possibility of further works on modelling the mechanical stress dependence of fluxgate sensors with stress-induced anisotropy.

However, it should be indicated that the proposed model is suitable only for modelling the uniaxial anisotropy of soft magnetic materials. Other models should be considered to model the other types of anisotropy observed experimentally, such as magnetocrystalline anisotropy of semi-hard or hard magnetic materials, or grain-oriented electrical steels.

## Data Availability

Not applicable.

## Supplementary Materials

The following supporting information can be downloaded at https://github.com/DKopala/3DPermeabilityTensor.
